# National vs. State Quarantine

**Published:** 1898-03

**Authors:** W. D. Travis

**Affiliations:** Covington, Ga.


### NATIONAL *VERSUS* STATE QUARANTINE.

BY W. D. TRAVIS, M.D.,

COVINGTON, GA.

It has been wisely said that the health of a nation is a national consideration involving international cooperation, and it should be as much a common cause for national defense as are our cherished liberties. When these are threatened by a foreign foe each State feels called upon to furnish men and means to be sent whenever and wherever the commander-in-chief of our nation shall direct. State rights are held in abeyance, while soldiers and sailors are rushed wherever common defense requires it; yet when "the pestilence that walketh in darkness and the destruction that wasteth at noonday," with weapons so tiny as to be invisible to the unaided eye, but still so powerful that armies and navies vanish away, and commerce, agriculture, manufacture and all of the industrial occupations, except that of the undertaker, are paralyzed, and even shotgun quarantine can not call forth respect for State lines, people say "the national government should have nothing to do with it; each State should manage its own quarantine."

Suppose during the Revolutionary War each colony had established a shotgun line of defense and kept its forces strictly within its own border, are you quite sure that we would now have a grand and glorious union boasting of its freedom? In united and concerted action there is strength, and this proves true in overcoming disease as well as other foes. Again, when an American citizen is imprisoned by a foreign power, nothing is said about State rights or from what State he comes. He is an American citizen, therefore entitled to all the aid and protection this government can give; but let some dread epidemic invade a town, and the lives of many people be endangered, not only from want of skill in treatment of disease, but also from ignorance of the best methods of extermination, experienced national aid is not wanted—its acceptance would be an infringement of State rights.

In some States, our own for example, there are no State boards of health; in others the rules and regulations are very meager, and are imperfectly carried out. By the establishment of a national health department, from which all politics are eliminated, we would have an executive authority that would be able not only to arbitrate, but dictate and command, where public health and safety demand it.

The subject of sanitary science is a complicated one, and should be carefully studied before any practical measures are instituted. Our sanitary system in its divisions should be modeled after our American government. As that has national, State, city and borough legislation with executive offices for each, so we should have legislation in sanitary matters for the nation, State, city and borough, with executive officers to enforce them. In ordinary cases the borough, city or State should be allowed to pass and execute their own sanitary laws; to protect their own local territory against the causes of disease localized within their own limits, but should not have the right to so regulate or neglect to regulate their local sanitation as to endanger neighboring States or distant governments. Our border should be entirely under the control and protection of a national sanitary authority. Our government should establish and maintain a perfect cordon of sanitary stations along our coasts, thoroughly furnished and equipped for the health and comfort of those under detention, and well supplied with all of the improved scientific appliances for fumigation and sterilization, under control of physicians eminent for skill in diagnosing and treating yellow fever, cholera, smallpox and other infectious diseases that are imported from other countries to our own. These officials should not be appointed by the President or be subject to political removal. They should be selected by medical associations or scientific bodies, and their term of office conditional upon their ability, efficiency and honest and faithful discharge of duty.

The State of Louisiana has one of the most perfect sanitary organizations in the world, as far as concerns quarantine against contagious diseases; for which credit is largely due to Dr. Holt. The whole country is indebted to this State for her system of quarantine; but is it just that Louisiana should bear, unaided, the heavy financial burden of guarding us against yellow fever? For she asserts that it is not a native of her soil, but is a foreign disease, which an efficient system of quarantine will keep out, and in the past she has boasted that she is fully able to protect her citizens from its ravages. But alas! where was this same confident boasting during the last epidemic of fever? She found herself not only unable to keep it out, but to stamp it out after it had entered, and was forced to appeal to the national government for aid. How the New Orleans quarantine is now regulated or may be regulated is something that concerns the whole country, and the care of guarding her gateway of disease should not be left to Louisiana alone. If her present system of quarantine can be improved or made more perfect by national supervision and aid, the government owes it to our country to supply that aid.

"The operation of a national department of health need no more interfere with or abrogate the rights and duties of the State boards of health to attend to the proper sanitation of their respective States than a national department of agriculture or a national code of laws need interfere or do away with a State department of agriculture or a State system of laws." If the government has power to establish an interstate commerce commission, and power to adopt measures to prevent the wrecking and robbing of railway trains, and thus assume jurisdiction over life and property in transit through different States, why has it not the right to try to prevent the migration of disease from one State or place to another? With the facilities that would be at its disposal, it should be the duty of the national board to be informed of the exact sanitary condition of every State in the Union and every country in the world, and to keep all sanitary authorities fully informed of the same. It should make a thorough study of epidemic diseases, and experts who have had all the advantages of an extended experience, aided by an improved medical science, should be immediately dispatched to any part of the Union to diagnose suspicious cases in order to arrest disease in its incipiency. Wise heads should meet and formulate a plan by which national, State, and city boards of health could work together, without friction, to save our country from scourges that have destroyed more lives during the last thirty years than the sword or the bullet during our civil war.

Lastly, it is in the interest of economy that we have a perfect system of national sanitation and quarantine. Dr. Albert L. Gihon says: "As the enlightened physician seeks to prevent his charges becoming ill, so should the guardian of the public health be able to forestall these emergencies, whose pecuniary cost in money expended and wasted, in trade paralyzed and diverted, in labor and its wages lost by the sick and terrified and dead, in a single epidemic, exceeds that of maintaining an efficient sanitary service for the whole country for a whole year. The fault of the medical profession has always been its lack of bold assertion of its rights; but it can no longer hesitate to declare to trade and commerce and agriculture and manufacture that the health and vigor which are essential to prosperity cannot be secured by their own unskilled, uninformed efforts." Then as epidemics rarely occur at the same time except in narrow limits, a few experts supplied by the general government would supersede the necessity of every State maintaining its own. Other expenses, too, could be saved, as, for instance, the government could easily establish, in places where most needed, plants for disinfection by means of formalin, now considered by the highest authorities the best disinfectant known. It would be utterly impracticable for each town to possess its own plant. Thousands of dollars and perhaps as many lives have been saved by our national weather bureau. Who can say how many lives and how much money might be saved by a national health department free from sectional bias and political environments whose motto should be, No North, no South, no East, no West, but my country and her interests.

---

### FOR CHRONIC CONSTIPATION.

℞ Tr. nux vom.......................................5 ʒ gtt. 8.

Tr. belladonnæ.......................................ʒ iiss.

Inf. sennæ.......................................ʒ v.

Inf. columbæ.......................................ʒ viiss.

M. Sig.: A teaspoonful before meals.

If the motions are drier than normal, a saline may be given at mealtime in addition to the above.—*Dr. C. B. Kelsey.*

